# B Cell Involvement in the Pathogenesis of Ankylosing Spondylitis

**DOI:** 10.3390/ijms222413325

**Published:** 2021-12-11

**Authors:** Rick Wilbrink, Anneke Spoorenberg, Gwenny M. P. J. Verstappen, Frans G. M. Kroese

**Affiliations:** Department of Rheumatology and Clinical Immunology, University of Groningen, University Medical Center Groningen, Hanzeplein 1, 9713 GZ Groningen, The Netherlands; a.spoorenberg@umcg.nl (A.S.); g.m.p.j.verstappen@umcg.nl (G.M.P.J.V.); f.g.m.kroese@umcg.nl (F.G.M.K.)

**Keywords:** B cells, B cell subsets, antibodies, autoantibodies, B cell infiltration, ankylosing spondylitis, axial spondyloarthritis

## Abstract

Extensive research into ankylosing spondylitis (AS) has suggested the major role of genetics, immune reactions, and the joint–gut axis in its etiology, although an ultimate consensus does not yet exist. The available evidence indicates that both autoinflammation and T-cell-mediated autoimmune processes are actively involved in the disease process of AS. So far, B cells have received relatively little attention in AS pathogenesis; this is largely due to a lack of conventional disease-defining autoantibodies. However, against prevailing dogma, there is a growing body of evidence suggestive of B cell involvement. This is illustrated by disturbances in circulating B cell populations and the formation of auto-reactive and non-autoreactive antibodies, along with B cell infiltrates within the axial skeleton of AS patients. Furthermore, the depletion of B cells, using rituximab, displayed beneficial results in a subgroup of patients with AS. This review provides an overview of our current knowledge of B cells in AS, and discusses their potential role in its pathogenesis. An overarching picture portrays increased B cell activation in AS, although it is unclear whether B cells directly affect pathogenesis, or are merely bystanders in the disease process.

## 1. Introduction

Spondyloarthritis (SpA) encompasses a heterogeneous group of related chronic rheumatic immune-mediated diseases, including axial spondyloarthritis (axSpA), which can be subdivided into ankylosing spondylitis (AS) and non-radiographic axSpA (nr-axSpA) [1]. AS is characterized by inflammation and structural damage of, predominantly, the sacroiliac joints and spinal vertebrae, including bone formation and bone loss, ultimately resulting in ankylosis, osteoporosis and vertebral fractures [2]. In contrast to AS, structural damage, as seen on X-ray, has not (yet) developed in patients with nr-axSpA. Besides the typical inflammation of the axial skeleton, AS patients may also develop peripheral manifestations, such as arthritis, dactylitis and enthesitis, as well as extra-skeletal manifestations (ESM) affecting the skin, gut, and eyes [3].

Despite increasing advancements in the therapeutic anti-inflammatory options for AS, the underlying pathogenesis is still largely unclear. [4]. According to current hypotheses, AS is both an autoinflammatory disease involving a wide variety of innate immune cells (e.g., neutrophils, macrophages, innate lymphoid cells) and an autoimmune disease with autoreactive lymphocytes [5]. The successful treatment of AS patients with TNFα and IL-17 inhibiting therapies illustrates the involvement of both the innate and the adaptive arms of the immune system in the disease process. All the available evidence indicates that the IL-23-IL-17 axis is pivotal in pathogenesis [6]. Biomechanical stress, especially at entheseal sites, seems to play an important role in the initiation of the disease in (genetically) susceptible individuals [7,8]. The extremely strong association (80–95% of patients) with the gene HLA-B*27, a class I surface antigen encoded by the B locus in the major histocompatibility complex (MHC), argues strongly for the central role of HLA-B27 in the pathogenesis of AS [9]. In addition to HLA-B27 and other MHC class I and II genes, genome-wide association studies (GWAS) identified a number of single-nucleotide polymorphisms in other genes as risk factors for AS. These genes, among others, involve genes in antigen processing, such as Endoplasmic Reticulum Associated Aminopeptidase 1 and 2 (ERAP1 and ERAP2) and genes directly or indirectly involved in the IL-23–IL-17 axis and Th17 and Th1 responses (including genes encoding for *IL-23R*, *IL-17A*, *IFNG)* [10,11,12].

One of the hypotheses is that the accumulation of misfolded HLA-B27 molecules in the endoplasmic reticulum (ER) results in ER stress and a sustained unfolded protein response (UPR) [13]. ER stress and UPR contribute significantly to excessive pro-inflammatory cytokine production, synergizing with pattern recognition receptor (PRR) stimulation [13]. The prominence of the MHC class I molecule HLA-B27 suggests the role of antigen presentation to CD8^+^ T cells and, thus, adaptive immune responses in AS pathogenesis. Arthritogenic peptide theory proposes the abnormal presentation of HLA-B27-restricted antigens to CD8^+^ cytotoxic T cells [14]. Furthermore, it is thought that intestinal dysbiosis, which has been demonstrated in AS patients, modulates the barrier function of the gut and activates immune cells [15]. The aberrant presentation of microbial antigens may result in the cross-reactivity to self-peptides, referred to as molecular mimicry. Finally, HLA-B27 features the ability to form homodimers, which can be expressed on the surface of cells. These homodimers may interact with killer-immunoglobulin-receptors expressed not only by NK cells, but also by CD4^+^ T cells, stimulating them to differentiate towards pathogenic Th17 cells [16]. Both Th1 cells and Th17 cells seem to play a role in the pathogenesis. Th17 cells are considered to be actively involved in the acute phase of inflammation in AS, whereas Th1 cells are suggested to perpetuate the inflammatory process [4]. The Th17 population is considered the main source of IL-17, a dominant cytokine in AS that is utilized as a therapeutic target [6]. Considering that the IL-23-IL-17 pathway can modulate bone metabolism, this pathway is of particular relevance in AS [17].

**Figure 1:**
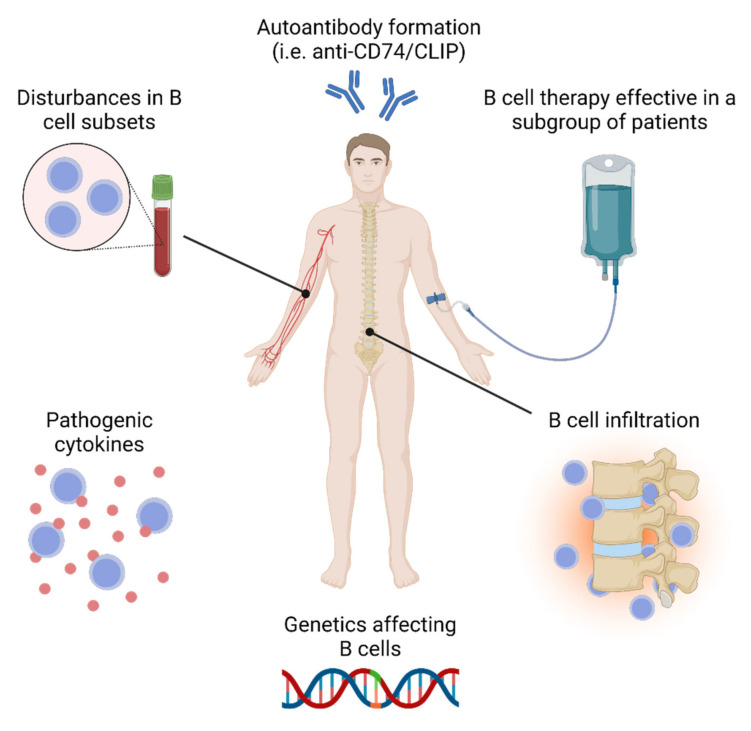
An overarching picture illustrating different aspects of B cell involvement in the pathogenesis of ankylosing spondylitis. This figure was created with BioRender.com (accessed on 9 November 2021).

Humoral immunity and, more specifically, B cells, have received relatively little attention over the past decades in the study of the pathogenesis of AS. This is largely due to the inability to demonstrate the presence of typical AS related autoantibodies. However, there are indications for B cell involvement in the disease pathogenesis of AS (Figure 1), demonstrated by the presence of genetic variants associated with B cell function, altered B cell subset distribution, associated (auto-) antibody formation, pathogenic B-cell-related cytokines, and the presence of B cell infiltrates in AS-affected inflammatory sites [18,19,20,21]. Furthermore, the depletion of B cells using rituximab (anti-CD20) in AS demonstrated promising results in at least a subcategory of patients naïve for anti-TNFα therapy [22]. In this paper, we review the current knowledge about the role of B cells in the pathogenesis of AS. Additionally, we discuss the potential use of B-cell-targeting therapies in AS.

## 2. Gene Variants in AS Modulating B Cell Function

There are no genetic associations found between genes that solely affect the function of B cells in AS patients. However, a number of genes are affected in AS; they can be expressed by B cells, thereby potentially influencing their functional role. In addition to the prominent role of the gene HLA-B*27, specific MHC class II alleles have also been associated with AS [23]. While MHC class I molecules are expressed on all nucleated cells, the expression of MHC class II molecules on nucleated cells is limited but is also present B cells. B cells are dependent for their full blown activation on CD4^+^ T cells. To this end, B cells present antigen to T cells in an MHC-class-II-mediated fashion. In AS, associations are found in the MHC class II loci *HLA-DPB1*, *HLA-DRB1*, and *HLA-DQB1* [23]. These associations are independent from HLA-B*27 since they are found both in HLA-B*27-negative and in HLA-B*27-positive AS patients [23]. This illustrates that besides the role of MHC class I, MHC class II genes are also involved in susceptibility to AS.

AS is also associated with variants (haplotypes) in the gene *BACH2* [12]. Initially, this gene was identified as a gene that encodes for an important transcriptional regulator in B cells [24]. For example, BACH2 is highly expressed in germinal center B cells and acts as an important negative regulator of Blimp-1, which is essential for plasma cell differentiation [25]. *BACH2* can, however, also be expressed by T-cells. Interestingly, BACH2 regulates T follicular helper cell (Tfh) formation and low BACH2 expression levels are needed to maintain a Tfh phenotype as well as the production of its signature cytokine, IL-21 [26,27]. Tfh cells and IL-21 are critically involved in germinal center formation and the differentiation of plasma cells. In summary, BACH2 is an important regulator of humoral immunity.

GWAS studies further identified the gene *TBX21* as a risk gene for the development of AS [12,28]. *TBX21* encodes for the transcription factor T-bet, which coordinates multiple aspects of both cellular and humoral immune responses [12,29]. T-bet is essential, for example, for Th1 cell differentiation, and promotes in these cells the production of Th1 cytokines (e.g., IFNγ, TNFα) and the expression of the inflammatory chemokine receptor CXCR3 [30,31]. In human B cells, T-bet expression has been implicated in class-switching from IgM towards IgG1 and IgG3 and the production of inflammatory cytokines [32,33]. Similar to T cells, the expression of CXCR3 on B cells is also regulated by T-bet [34]. We have shown in AS patients that amongst CD21^low^ B cells (see following section), the frequency of CXCR3^+^ B cells is indeed strongly associated with the frequency of T-bet^+^ B cells [20]. T-bet^+^ B cells are postulated to represent germinal center-derived B cells destined to become antibody secreting cells [33]. Increased frequencies of T-bet-expressing B cells are described in conditions of persistent stimulation, such as viral infections and autoimmunity [33].

Finally, variations in the gene encoding for IL-10 have also been found in AS patients [35]. This immune response regulatory cytokine is produced by both regulatory T and B (CD24^+^CD38^+^) cells [36,37]. Chen et al. demonstrated that regulatory B cells from AS patients produce less IL-10 than non-AS control individuals and are defective in the suppression of CD8^+^ T cell activation [38]. Whether this defect can be (partly) attributed to certain haplotypes is not known.

Taken together, several AS-associated genes have been identified that potentially affect B cell function. To what extent these genetic variations are involved in AS pathogenesis remains to be elucidated.

## 3. Disturbances of the Peripheral B Cell Compartment in AS

Recent decades provided continuous advancements in knowledge regarding cellular dynamics and effector functions of various B cell populations, thereby shedding more light on the mechanisms of underlying autoimmune and autoinflammatory disease [39]. The involvement of B cells in the pathogenic processes of these various chronic immune-mediated diseases is reflected by disturbances in the subset distribution of peripheral B cell compartment [40]. Furthermore, in AS, the composition of B cell subsets circulating in the blood is altered compared to healthy individuals. In this section, we summarize the reported changes in the peripheral B cell compartment seen in AS patients (see Table 1).

### 3.1. Disturbances in the Total B Cells and Major B Cells in Subpopulations

In most studies, normal frequencies of total circulating B cells (CD19^+^) were seen in AS patients [20,38,41,42,43,44,45]. In these studies, disease activity was not taken into consideration. However, when disease activity was taken into account, two studies reported elevated frequencies of total B cells [46,47]. Lin et al. demonstrated that AS patients with active disease exhibited higher frequencies of total B cells in their blood, compared with patients with stable disease and healthy individuals [46]. In this study, active disease was defined by the following criteria: visual analogue scale of the duration and severity of morning stiffness (≥30) and positivity for two out of three other disease activity-related scores (patient’s global assessment, global back pain and nocturnal back pain, Bath Ankylosing Spondylitis Functional Index). In addition, higher frequencies of total B cells were shown in patients with both peripheral (not further specified) and axial involvement in comparison to those with axial involvement only and healthy individuals. Importantly, when taking all the (active and not-active) patients together, significant positive associations were found between the frequency of total B cells and several clinical parameters related to disease activity, including the Bath Ankylosing Spondylitis Disease Activity Index (BASDAI) score, global back pain and nocturnal back pain [46]. Another study that demonstrated elevated frequencies of total circulating B cells in AS patients included exclusively patients with active disease (ASDAS ≥ 1.3) [47]. These authors were, however, unable to confirm a relationship between B cell frequencies and clinical parameters. A plausible explanation for the discrepancy with the study of Lin et al. might be that the latter study included exclusively active disease patients.

Furthermore, with respect to the distribution of major B cell subpopulations (i.e., transitional, naïve and memory B cell subsets) present in the blood of AS patients, data are limited and not conclusive. As demonstrated in Table 1, no consistent differences were seen in the frequencies of transitional (CD24^hi^CD38^hi^), naïve (CD27^−^IgD^−^) and memory (CD27^+^IgD^−^) B cell subpopulations compared to healthy individuals [38,45,47].

**Table 1:** Studies on AS patients presenting frequencies of total number of B cells and B cell subsets compared to healthy individuals.

Reference	Total B	Transitional	Naïve	Memory	Plasmablast/Cell
	CD19^+^	CD24^+^CD38^+^	IgD^+^CD27^−^	IgD^−^CD27^+^	CD27^hi^/CD38^hi^
Brand et al. [41]	Normal	N/A	N/A	N/A	N/A
Chen et al. [42]	Normal	N/A	N/A	N/A	N/A
Szanto et al. [43]	Normal	N/A	N/A	N/A	N/A
Bautista-Caro et al. [48]	N/A	N/A	N/A	N/A	↓ ^$^
Lin et al. [46]	↑ *	Normal	Normal	Normal	↑ **
Long et al. [44]	Normal	N/A	↑ *	↓ *	↑
Yang et al. [47]	↑	↓	N/A	↑/↓ ^%^	↓
Bautista-Caro et al. [45]	Normal	↑	N/A	N/A	N/A
Chen et al. [38]	Normal	Normal	↓	↑	↑
Niu et al. [49]	N/A	N/A	Normal	↓	N/A
Wilbrink et al. [20]	Normal	Normal	Normal	Normal	↑

A significantly higher frequency of the B cell populations is indicated as ↑ and a lower frequency with ↓. ^$^ Only in AS patients naïve for TNF blockers. * Only in AS patients with active disease (different definitions used), ** only in AS patients with peripheral and axial involvement. ^%^ Significant increase in switched memory B cells (CD19^+^CD27^+^CD38^−^IgD^−^IgM^−^) and decrease in memory B cells (CD19^+^CD24^+^CD27^+^CD38^+^IgD^+^IgM^+^) and non-switched memory B cells (CD19^+^CD24^−^CD27^+^CD38^+^IgD^+^IgM^+^). N/A: not assessed.

### 3.2. IL-10 Producing B Cells in AS

Regulatory B cells encompass a relatively small subset of B cells defined by their signature cytokine IL-10, which is primarily known for its anti-inflammatory properties. Most regulatory B cells have a cell surface phenotype that is shared with transitional B cells (CD24^hi^CD38^hi^). In AS, most circulating IL-10 producing B cells display a transitional phenotype (CD24^hi^CD38^hi^) and a smaller fraction of IL-10 producing B cells is represented by plasmablasts [38]. Although the frequencies of circulating CD24^hi^CD38^hi^ B cells are comparable to healthy individuals, ex vivo cultures of purified regulatory (CD24^hi^CD38^hi^) B cells showed that spontaneous IL-10 secretion was lower for AS patients. Ex vivo B cell receptor plus CD40 stimulation of these cells lead to increased IL-10 secretion, although the IL-10 levels produced by the regulatory B cells from AS patients never reached the levels obtained by healthy regulatory B cells. Whether the frequencies of IL-10-producing circulating B cells are altered in AS patients was not explored in this study.

The cell surface protein CD5 can be expressed by multiple B cell subpopulations, including B cells with a (CD24^hi^CD38^hi^) transitional phenotype [50]. In humans, the expression of CD5 is, at least in part, associated with B cell activation [51]. CD5-expressing B cells in SpA patients (74% AS patients) and healthy individuals are enriched in IL-10 production compared to CD5- B cells [52]. Increased frequencies of CD5^+^ B cells were observed in blood from SpA patients, compared to RA patients and healthy individuals [52]. Whether this also implies that there are more IL-10-producing cells in blood from AS patients, compared to controls, is not known. Notably, data regarding the serum levels of IL-10 in AS patients do not provide clear indications with respect to variations in IL-10 levels, as results are contradictory [53,54]. Altogether, although phenotypic analysis shows that frequencies of regulatory B cells in AS patients are not reduced, their function in terms of IL-10 production might be impaired.

### 3.3. Activated B Cells in AS

B cell activation is associated with the increased expression of several cell surface molecules, including CD86, CD38 and CD95 [55,56,57]. CD86 is important for the co-stimulation of T cells, which provide help to B cells, whereas CD38 features multifunctional properties and is essential, among other things, for the regulation of intracellular Ca^2+^ levels [50,55,56]. The “death receptor” CD95, also known as Fas receptor, plays a role in the apoptosis of cells and removal of exhausted and/or autoreactive B cells [57,58]. In patients with AS, higher frequencies of B cells are observed expressing the co-stimulatory cell surface molecule CD86 or the pro-apoptotic receptor CD95 [49]. Frequencies of CD95^+^ B cells in AS patients are positively correlated with BASDAI score, which suggests a rise in the number of activated B cells in patients with more active disease. Although the proportion of B cells expressing CD38 were similar in AS patients and controls, the frequencies of CD38-expressing B cells were similarly associated with BASDAI score, as noted for CD95^+^ B cells. In particular, plasmablasts (and plasma cells) express high levels of CD38, along with high levels of CD27 [59]. In AS patients, the majority of studies (four out of six), including our own, revealed increased frequencies of circulating plasmablasts (CD27^hi^/CD38^hi^ cells) [20,38,46]. Thus, phenotypical analysis of circulating B cells in AS provided some evidence that there are more activated B cells and plasma cells, compared to in healthy individuals.

B cell activation largely depends upon the activity of Tfh cells, defined as CD4^+^CXCR5^+^ T cells. In AS patients, analyses of circulating Tfh cells frequencies yield contradictory results, with two out of three studies reporting elevated proportions [44,48,60]. The frequencies of circulating Tfh cells seem to correlate positively in AS patients’ blood with the frequencies of CD38^+^ B cells and plasmablasts [44,48]. Thus, not only do activated B cells and plasmablasts appear to be increased in AS patients, but also, most likely, the subset of CD4^+^ T cells critically involved in their activation.

### 3.4. The CD21^low^ B Cell Population in AS

Recently, a particular B subset characterized by low expression of CD21, a co-stimulatory molecule important for B cell activation, gained more attention because of its association with chronic inflammation and autoimmunity [61]. High levels of circulating CD21^low^ B cells are found in systemic lupus erythematosus (SLE) [62], primary Sjögren syndrome (pSS) [63], rheumatoid arthritis (RA) [64], and common variable immunodeficiency [64]. CD21^low^ B cells are found among naïve as well as switched and unswitched memory B cells [61]. CD21^low^ cells are generally considered to be the result of chronic antigenic stimulation and/or defective negative selection of autoreactive cells [64,65]. Furthermore, in AS patients, higher levels of CD21^low^ B cells were observed [38]. Recently, we also analyzed the frequency and phenotype of CD21^low^ cells in patients with axSpA, of whom most (80%) were diagnosed with AS [20]. Since transitional (CD38^hi^) B cells express low levels of CD21 [61], we excluded CD38^hi^ cells from our analysis. We observed that axSpA patients indeed exhibited higher frequencies of CD21^low^ B cells compared to healthy individuals, and extended this observation by showing that this was largely due to an increase in the frequency of CD27-negative CD21^low^ B cells (CD27^−^CD38^low^CD21^low^ B cells) [20]. The frequencies of CD27-positive CD21^low^ B cells (CD27^+^CD38^low^CD21^low^ B cells) were not affected. In our study, a similar increase in CD27^-^CD38^low^CD21^low^ B cells was seen in patients with pSS, a typical B-cell-mediated systemic autoimmune disease. A small fraction (up to 22%) of the CD27^-^CD38^low^CD21^low^ B cells in axSpA patients were isotype-switched B cells (i.e., IgD^−^IgM^−^) [20]. Interestingly, the vast majority of these isotype-switched CD27^-^CD38^low^CD21^low^ B cells (but not the unswitched CD27^-^CD38^low^CD21^low^ B cells) co-expressed the transcription factor T-bet and the integrin CD11c in both axSpA and pSS patients and healthy individuals [20]. Similarly, elevated numbers of T-bet^+^CD11c^+^B cells, which are largely negative for CD21, are detected in SLE [66]; in this B-cell-mediated disease, these cells are thought to be the precursors of antibody-secreting cells [67,68]. This is of relevance to AS, as we also observed an increase in circulating plasmablasts. Compared to healthy individuals, the frequency of CXCR3-positive CD27^−^CD38^low^CD21^low^ B cells was significantly higher in the axSpA patients, pointing towards an increased capacity to migrate to sites of inflammation [20]. Furthermore, the patients with a history of ESM displayed the highest frequencies of circulating CD27^−^CD38^low^C21^low^ B cells. The frequency of CD27^-^CD38^low^CD21^low^ B cells also correlated positively with the clinical parameters, age and erythrocyte sedimentation rate. Despite the absence of clear associations between CD27^-^CD38^low^CD21^low^ B cells and disease activity scores (i.e., ASDAS) in AS, it is tempting to speculate that in AS switched, these CD27^-^CD38^low^CD21^low^ B cells, most of which express T-bet and CD11c, are involved in the disease process. Whether they also reflect a pool of antibody-secreting cell precursors in AS needs to be established.

In summary, the analysis of circulating B cells and their subsets portrays an overarching picture of increased frequencies of activated B cells and plasmablasts in AS patients. This is substantiated by the finding that their frequencies are associated with higher states of disease activity. Nevertheless, there are differences between the studies, which might be in part due to the method of B cell analysis (including the definition of B cell subsets) as well patient characteristics and disease state.

## 4. Auto-Reactive and Non-Autoreactive Antibodies in AS

**Figure 2:**
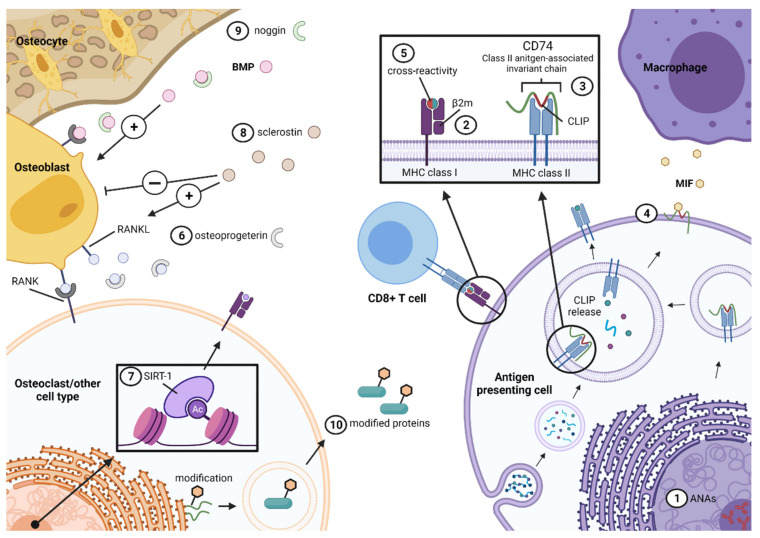
An overview of various auto-reactive and non-autoreactive antibody targets found in ankylosing spondylitis (AS), ranging from ① anti-nuclear antibodies (ANAs) to nuclear constituents. Additionally, autoantibodies to regulators and proteins important in antigen presentation are observed. Antibodies to the light chain ② beta-2 microglobulin of MHC class I molecules have been found. Furthermore, autoantibodies to ③ MHC class II invariant chain, known as CD74 (green), including its selective domain CLIP (red), which is bound to the groove of MHC class II molecules, have been observed. In addition, ④ CD74 may be membrane bound and act as a receptor for macrophage migration inhibitory factor (MIF). Besides the presence of antibodies reactive to antigen presenting molecules, antibodies towards ⑤ microbial agents are found, that display the potential to cross-react (red) with self-antigens (green). Furthermore, autoantibodies to regulators of bone metabolism are observed in AS, such as ⑥ osteoprogeterin, which negatively regulates of RANKL, ⑦ SIRT-1, a primarily nucleated enzyme that deacetylates transcription factors, ⑧ sclerostin, an inhibitor of bone formation, mainly produced by osteocytes, and ⑨ noggin, a protein antagonizing the bone morphogenetic protein (BMP) signalling pathway. Lastly, the formation of auto-reactive antibodies was detected towards ⑩ proteins that underwent post translational modification. Next to the aforementioned antibodies, unidentified targets have also been observed, such as reactivity to UH-axSpA peptides. This figure was created with BioRender.com (accessed on 9 November 2021).

Antibody production and secretion is the defining hallmark of B cell function. The involvement of B cells in the pathogenesis of diseases can therefore be reflected by the presence of specific (sets of) antibodies involved in the initiation and/or perpetuation of the disease. Although disease-defining (auto-) antibodies have not (yet) been identified in patients with AS, there is accumulating evidence of the presence of specific (auto-) antibodies enriched in, or associated with, these patients. Here, we provide an overview of various autoreactive and non-autoreactive antibodies found in AS (Table 2 and Figure 2).

**Table 2:** Autoreactive and non-autoreactive antibodies in AS.

Reactivity Against	Category	Reference
**Intracellular components**		
Nuclear antibodies (ANAs)	Nuclear antigens	[69,70]
Antineutrophil cytoplasmic antibodies(ANCA)	Cytoplasmic molecules	[71,72]
Prefoldin subunit 5	Chaperone proteins	[73]
Beta-2 microglobulin and CD74	MHC class I and II related molecules	[74,75,76,77,78,79,80,81,82]
**Microbial components**		
*Klebsiella pneumonia*, and mycobacterium	Microbial epitopes and molecules	[83,84,85,86,87,88,89]
**Bone metabolism and connective components**		
Sclerostin, bone metabolism, and signaling molecules (NAD-dependent protein deacytelase sirtuin-1 and osteoprotegerin)	Bone tissue	[90,91,92,93,94,95,96]
Extracellular matrix proteins, collagen and ATP synthase subunit-α	Connective and skeletal muscle tissue	[92,97,98]
**Post-translational modifications**		
Citrullinated cyclic peptides and carbamylated proteins	Modified molecules	[99,100,101]
**Other components**		
UH-axSpA peptides	Unidentified proteins and peptides	[102]

For a more elaborate overview on antibodies in AS, especially against microbial components, we refer to a review by Quaden et al. [22].

### 4.1. Autoantibodies Reactive to Intracellular Antigens

A characteristic feature of systemic auto-immune diseases, such as SLE, pSS and systemic sclerosis, is the presence of antibodies directed against intracellular components including anti-nuclear and anti-cytoplasmic constituents. Furthermore, in patients with AS, several studies revealed the presence of anti-nuclear antibodies (ANAs), although the frequencies of patients with ANAs varied significantly between the studies (6–60%) [69,70,103,104]. Differences in methodology, patient cohorts and biological therapy use may explain at least some of the variation found in these ANA frequencies [105]. When looking at cytoplasmic molecular targets, three studies described the presence of anti-neutrophil cytoplasmic antibodies (ANCAs) in a relatively small proportion (14–20%) of AS patients [71,72,104]. Thus, while antibodies against cellular components have been demonstrated in a subgroup of AS patients, the sensitivity of ANAs and ANCAs for AS remains unclear.

In addition to these types of antibodies, traditionally associated with systemic autoimmune diseases, antibodies to the intracellular protein prefoldin subunit 5 (PFDN5) have also been found in a subgroup of AS patients [73,87]. PFDN5 is a chaperone protein that is involved in stabilizing polypeptides and protecting cells from aggregated protein-induced cell death [106]. PFDN5 is thought to play a protective role in the apoptosis of retinal cells [73]. Significantly elevated levels of PFDN5 antibodies and free PFDN5 were found in AS patients with uveitis compared to AS patients without this manifestation and non-AS patients [73]. The functional meaning of these anti-PFDN5 antibodies is not clear yet, and the potential value of these antibodies as a biomarker for risk of uveitis in AS patients warrants further investigation.

### 4.2. Antibodies Directed against Intracellular Molecules Involved in Antigen Presentation

The correctly folded form of HLA class I molecules assembles with beta-2 microglobulin (β_2_m), which is the light chain of class I molecules, and a peptide [107]. These stabilized trimers are expressed on the cell surface and present antigenic peptides to CD8^+^ T cells. When surface MHC class I molecules are degraded, β_2_m is released into the serum. Elevated levels of free, soluble serum β_2_m have been associated with chronic inflammatory conditions, including AS, SLE, and RA [74]. Autoantibodies to β_2_m have also been found in AS patients, at frequencies similar to those in patients with SLE (68% and 71%, respectively), which are much higher than those in RA patients (27%) and healthy controls (5%) [74]. Whether anti-β_2_m antibodies are a consequence of chronically elevated free β_2_m levels is not known, nor is it known whether these antibodies contribute to the pathogenesis of the disease.

One of the most intriguing autoantibodies detected in AS are antibodies directed to the MHC class II histocompatibility antigen invariant (gamma) chain, also known as CD74 [75,76,77,78,79,80,81,82]. This invariant chain is involved in preventing the premature binding of peptides to the peptide-binding groove in MHC class II [108]. Blocking of the groove is carried out by a small fragment of the CD74 molecule, called CLIP (class II associated invariant chain peptide). In addition to its role in antigen processing, CD74 is also a high-affinity receptor for the pro-inflammatory cytokine macrophage migration inhibitory factor (MIF) [109]. MIF is produced by a wide variety of innate and adaptive immune and non-immune cells [110]. MIF is implicated in several biological functions and may also modulate bone metabolism by promoting both osteoclastic and osteoblastic activity [111,112,113,114,115]. Binding of MIF to CD74 on B cells may induce survival, proliferation, and maturation via NF-κB mediated transcription [116,117,118]. In AS patients, MIF serum levels are significantly elevated, along with higher levels of MIF in synovial fluid [114,119]. In addition, serum MIF levels were significantly increased in AS patients with radiographic progression of the disease compared to non-progressors [114]. Several studies revealed the presence of anti-CD74 antibodies in patients with axSpA and its phenotypes [75,76,77,78,79,80,81,82,120]. However, the prevalence of anti-CD74 antibodies varied considerably between the studies, and the following percentages of IgG anti-CD74 antibodies were reported for different axSpA patient populations: axSpA patients (80–92%), AS patients (40–80%), early axSpA (46%), and nr-axSpA (17%) [75,77,78,120]. In comparison, 5–44% of the healthy individuals demonstrated anti-CD74 antibodies in their serum. In addition to IgG anti-CD74 antibodies, IgA anti-CD74 antibodies can also be detected in 23–50% of axSpA patients and in 47% of non-radiographic axSpA patients [76,78,80,81,82]. The presence of serum IgA anti-CD74 antibodies is associated with radiographic progression in the axial skeleton, sacroiliitis on MRI, and heel enthesitis [76,78,82].

The detection of IgG autoantibodies in the functionally active peptide of CD74, known as CLIP, seems to be more specific: 85–97% in axSpA patients exhibit anti-CLIP antibodies, in contrast to only 1% of healthy individuals, with a reported specificity of 92% [75,79]. The detection of anti-CD74/CLIP autoantibodies by ELISA is challenging, and the standardization of assays is highly warranted [75]. In addition to differences between the study populations, these technical difficulties may explain at least part of the variation in the prevalence of antibodies in CD74/CLIP seen in different studies.

Why anti-CD74 antibodies are formed in AS patients is not known. However, recently, van Kempen et al. found that monocytes in a subset of AS patients exhibit a defect in the enzyme signal peptide peptidase-like 2A (SPPL2A) [121]. Reduced SPPL2A activity causes the accumulation of CD74 and CD74 degradation products, which are deposited on the surface membrane upon exposure to IFNγ stimulation [121]. These degradation products can be recognized by the anti-CD74 antibodies in patients’ sera. The reason for the diminished functioning of SPPL2A in AS patients is not known. This aberrant expression of CD74 fragments might, however, be a plausible explanation for the induction of anti-CD74 autoantibodies in AS patients. How the anti-CD74 antibodies contribute to the pathogenesis of AS, and whether these antibodies mimic (agonistic) or block (antagonistic) MIF effects on the CD74 expressing cells (including osteoclasts and B-cells), is not clear yet.

### 4.3. Antibodies Affecting Bone Metabolism

Systemic loss of bone, along with the formation of new bone, is a classic feature of AS [17]. Although it is not completely known via which mechanisms bone remodeling and repair are affected in AS, the presence of serum (auto-) antibodies in a wide variety of connective and musculoskeletal tissue-derived proteins has been identified [92,97,98]. For example, a small proportion (8%) of axSpA patients exhibits autoantibody reactivity to osteoprotegerin (OPG) [96]. The prevalence of anti-OPG antibodies is similar in RA patients (8%), but is only observed in 1% of the healthy population [122]. OPG is a protective factor, preventing excessive bone resorption by binding RANKL (receptor activator of nuclear factor kappa-Β ligand), which functions as a key factor in osteoclast differentiation and activation [123]. RANKL is typically membrane-bound [124], but is also found in secreted form and is produced by various cell types, including osteoblasts, osteocytes, T cells, and even B cells. Among B cells, most RANKL is expressed by switched memory B cells [125]. Anti-OPG antibodies prevent the interaction of OPG with RANKL, leading to sustained activity of osteoclasts, resulting in bone loss. This mode of action of anti-OPG antibodies, illustrated in axSpA patients as the presence of anti-OPG antibodies, is independently associated with lower hip bone mineral density and a previous history of fractures (vertebral and non-vertebral), but not with lumbar spine bone mineral density [96]. Although no association of anti-OPG antibodies with serum levels of bone turnover markers and serum RANKL has been observed, the presence of these antibodies may contribute to detrimental bone loss in a subcategory of axSpA patients. This finding is particularly relevant for AS, considering that the prevalence of osteoporosis is high (25%) among these patients [126]. The detection of these antibodies may therefore be of value as a risk indicator of hip bone mineral density loss and hip fractures.

Autoantibodies can also be found in other important regulators of bone homeostasis, such as NAD-dependent protein deacetylase sirtuin-1 (SIRT1), sclerostin, and noggin. SIRT1 is an intracellular enzyme, mainly found in the nucleus, that regulates transcription factors affecting cell metabolism, apoptosis, autophagy, differentiation and immune functions [127]. SIRT1 also plays a pivotal role in bone homeostasis and stimulates osteogenesis, as demonstrated in SIRT1-deficient mice that displayed a dramatic decrease in bone mass [128]. This effect of SIRT1 is possibly mediated by sclerostin. SIRT1 is a repressor of the sclerostin gene, which is an inhibitor of bone formation [127]. In the absence of SIRT1, sclerostin is not inhibited, resulting in reduced bone formation and a net loss of bone. In axSpA, the prevalence of serum IgG antibodies compared to SIRT1 is 19%, which is similar to the prevalence in RA patients, but much higher compared to psoriatic arthritis (PsA) patients (1%) [93]. Additionally, in comparison to healthy individuals, anti-SIRT1 antibody titers were significantly increased in patients with AS. Interestingly, serum levels of anti-SIRT1 antibodies were particularly elevated in AS patients with early hip involvement of less than one year compared to AS patients without hip involvement or hip involvement more than a year previously [93]. In addition, anti-SIRT antibody titers were significantly increased in females, although in males, the levels of anti-SIRT antibodies were still higher than in healthy individuals [93]. Unfortunately, the relationship between levels of (axial) bone mineral density was not investigated in this study. How antibodies to SIRT1, an intracellular regulator, may exert their effect in AS patients, and whether this is mediated by sclerostin, is not clear.

Furthermore, IgG autoantibodies to sclerostin and noggin have been observed in the serum of AS patients [91]. Sclerostin is a secretory glycoprotein, mainly produced by osteocytes; it is believed to inhibit the Wnt/β-catenin pathway, resulting in suppressed bone formation [91,129]. Noggin is a polypeptide secreted by a wide range of cell types, negatively affecting bone formation by antagonizing the bone morphogenetic protein signalling pathway [130]. Lower levels of sclerostin and noggin may thus relatively enhance bone formation. However, data on serum sclerostin levels in AS patients compared to healthy individuals are not consistent, and noggin serum levels are, to our knowledge, only reported by one study [91,131]. In AS patients, relatively low serum sclerostin levels have been associated with radiographic progression, as illustrated by the formation of new syndesmophytes [131,132]. Additionally, lower sclerostin levels, along with higher anti-sclerostin titers, predict the presence of axSpA in patients with inflammatory bowel disease (IBD) [90]. The overexpression of noggin was shown to prevent the progression of ankylosing enthesitis in an experimental mouse model [133]. Whether autoantibodies against sclerostin or noggin lead to increased bone formation in AS patients warrants further investigation. The presence of autoantibodies may contribute, at least partly, to lower sclerostin or noggin levels. The sensitivity and specificity of autoantibodies to sclerostin (74%, 57%) and noggin (68%, 68%) versus individuals with mechanical back pain is substantial [91]. Interestingly, the absence of sclerostin, as demonstrated in sclerostin knock-out mice, adversely impacts B cell development and survival [134]. This effect is possibly mediated through the reduced expression of the chemokine CXCL12 produced by bone marrow stromal cells [135].

Altogether, despite emerging data suggestive of autoreactivity against proteins affecting bone homeostasis, the questions of why and how these autoantibodies are formed and contribute to the imbalance of bone formation and pathogenesis in AS patients remain to be answered.

### 4.4. Antibodies to Cross-Reactive Microbial Antigens

Patients with AS may experience increased permeability of the intestinal tract, also referred to as a ‘leaky’ gut [136]. In line with this notion, a substantial proportion of the AS patient population displays subclinical inflammation of the gut and even develops inflammatory bowel disease [3,137,138]. The impairment of the protective function of the bowel allows enhanced interactions between cells of the immune system and microbes. Since IgA is the dominant class of immunoglobulins at the mucosal lining of the digestive system, the elevated IgA levels found in the serum of AS patients may reflect intestinal involvement [139,140]. The reason for the reduced mucosal barrier function is not known, and whether the altered microbiota composition in the gut of AS patients is the cause or consequence of the disease also remains to be shown [141]. In view of these findings, the presence of serum antibodies in a wide variety of (intestinal) microbial antigens in AS patients might not be surprising (see the review by Quaden et al.) [21]. Here, we briefly describe only a few antibodies in AS patients to microbial antigens that may cross-react with self-antigens.

In recent decades, the presence and function of serum IgA and IgG antibodies against *Klebsiella pneumonia* has been extensively investigated in patients with AS, demonstrating the higher serum levels of these antibodies in AS patients compared to healthy controls [83,88,89]. A more recent study using a peptide library demonstrated, strikingly, that serum IgG antibodies against a peptide of *Klebsiella pneumoniae*-derived dipeptidase protein (DPP) sequence were prevalent in nearly all (95%) patients with AS; by contrast, they were present in none of the healthy individuals and in only 1–1.5% of patients with RA and PsA [86]. Interestingly, the 5% of AS patients not displaying anti-DPP IgG antibodies in their serum were negative for HLA-B*27 [86]. This DPP epitope shares similarities with the self-proteins present within fibrocartilaginous tissue, including various types of collagen [86]. These findings may suggest that molecular mimicry of *Klebsiella pneumoniae*-derived peptides and connective tissue-derived proteins contributes to the activation of autoreactive T and B cells, particularly in HLA-B*27-positive individuals. Another example of the involvement of molecular mimicry in AS pathogenesis is the heat shock proteins on *Mycobacterium* that cross-react with self HSPs. In AS patients, but also in patients with other autoimmune diseases, such as RA and pSS, serum IgG antibodies to *Mycobacterium* HSP65 are significantly elevated compared to controls [87,142]. These antibodies cross-react with human HSP65, but the functional consequences of these anti-HSP65 antibodies, as well as the anti-DPP antibodies for the initiation and/or perpetuation of AS, are not understood.

### 4.5. Antibodies Directed against Modified Self-Proteins and Self-Peptides

Following protein biosynthesis, a common mechanism that modulates their structural and functional properties is post-translational modification, including citrullination and carbamylation. Microbial virulence factors may contribute to the generation of these modified proteins, secondary to inflammation, as described in RA [143]. An accumulation of these modified proteins may evoke an (auto-) immune response, resulting in the formation of anti-citrullinated protein antibodies (ACPA) and anti-carbamylated proteins (anti-CarP) antibodies [143]. In marked contrast with RA, the prevalence of these antibodies in the serum of AS patients is very low (2–4% for ACPAs and 10–20% for anti-CarP antibodies). Therefore, the diagnostic/prognostic value of ACPAs and anti-CarP antibodies is also rather limited in AS, compared to RA [99,100,101,144].

A study investigating novel (auto-) antibodies to self-peptides in axSpA patients used a complementary DNA phage library from the synovial hip tissue of axSpA patients to screen early axSpA patients for serum IgG antibodies directed against peptides derived from this library [102]. Antibody reactivity was observed in nine as-yet unidentified peptides (UH-axSpA), among which three UH-axSpA peptides showed the most potential [102]. The combined presence of these three UH-axSpA peptides was significantly increased in early-axSpA patients (14%) compared to controls with chronic lower back pain (5%); the post-test probability increased to 91% when adding CRP levels and HLA-B27 positivity [102]. However, the clinical relevance of antibodies directed to these UH-axSpA peptides, and their diagnostic value, requires further examination.

In summary, antibodies to a wide variety of autoantigens, cross-reactive microbial antigens and modified self-proteins have been detected in patients with axSpA and/or AS. Although the sensitivity and specificity of these (auto-) antibodies is generally low, they may play a role in pathogenesis and may well reflect the involvement of B cells in the disease process.

## 5. B-Cell-Associated Cytokines in AS

The IL-23/IL-17 pathway is considered to play a pivotal role in the pathogenesis of AS and SpA-related disease [145]. Several studies demonstrated higher levels of serum IL-23 and IL-17 in patients with AS [146,147,148]. Over the past few years, the IL-23/IL-17 axis has been targeted in clinical trials using monoclonal antibodies to suppress these cytokines [6]. Currently, the inhibition of IL-17 yields the most promising results, especially in patients naïve for treatment with TNF inhibitors. Unexpectedly, IL-23 blockade for the treatment of AS was inefficacious [149,150]. A reason for this lack of effect is unclear, but apparently IL-23-independent IL-17 pathways are (also) involved in the disease process [151,152]. Of relevance for AS, IL-23, and IL-17 possess immune functions and effects on bone homeostasis, and seem to contribute to systemic bone loss and local bone formation [17].

As mentioned previously, potent sources of IL-17 are innate immune cells and T cells [153]. Current data suggest that a variety of T cells, including Th17 cells, MAIT cells, and colonic, but not enheseal, γδ T cells, may contribute IL-17 production in SpA [154,155,156,157,158]. In addition to T cells, B cells are also able to produce and secrete IL-17, as shown in RA and healthy individuals [159,160]. Most of the IL-17^+^ B cells (54%) in the blood of healthy individuals are part of the naïve (CD19^+^CD27^−^IgD^+^) B cell pool, although a substantial fraction (21%) exhibits a transitional/regulatory phenotype (CD24^hi^CD38^hi^) [160]. In addition to its role in IL-17 production, IL-17 exerts modulatory effects on B cell function and IL-17 promotes B cell proliferation, differentiation, and plasma cell generation, and the influences migration of B cells [161,162,163]. Furthermore, IL-17 signalling is proposed to orchestrate the formation of (ectopic) lymphoid tissue, thereby contributing to (auto-) antibody production [161,164]. Whether B cells are also a contributing cellular source for IL-17 in AS patients is not yet known.

IL-21 is a cytokine that is critically involved in memory B cell and plasma cell formation [165]. Two studies revealed that the serum and plasma levels of IL-21 are elevated in AS patients, compared to healthy individuals [166,167]. IL-21 is mainly produced by Tfh cells and Th17 cells [165]. The frequencies and numbers of both T cell subsets are indeed increased in patients with AS [158,167]. IL-21 upregulates the expression of T-bet and CD11c by B cells, which are thought to be precursors of antibody-secreting cells [67,68,168]. The higher frequency of plasmablasts present in the peripheral blood of AS patients may well reflect the enhanced IL-21-driven activity of B cells in AS [20].

## 6. B Cell Infiltration of AS Target Tissues

Typically, chronic inflammation is associated with the infiltration of lymphocytes, including B cells, in addition to a wide variety of non-lymphoid cells. Infiltrated B cells have been observed at classical sites of inflammation in AS, such as the spine and the sacroiliac joints [18,19]. The first immunohistochemical study evaluating the presence of B cells (CD20^+^) in the axial skeleton of AS patients examined biopsies from zygapophyseal joints [19]. The number of B cells was significantly elevated in the biopsies of AS patients with persistent inflammation compared to patients without inflammation and controls [19]. Moreover, in AS patients, B and T cells were segregated from each other within the lymphoid aggregates, indicative of ectopic lymphoid tissue. Given the role of Th17 cells and IL-17 in AS, it is not surprising that this ectopic lymphoid tissue is formed [161]. Peng et al. demonstrated that in the sacroiliac joints, large amounts of (CD20^+^) B cell infiltrates were observed in the bone marrow and fibrous tissue of patients with AS and nr-axSpA [18]. In line with these results, a study on transgenic mice that overexpressed transmembrane TNF, which induces both axial and peripheral SpA features, demonstrated lymphoid aggregates in spinal bone marrow, in addition to lymphocytic infiltration along ligaments in connective tissue at the border intervertebral discs [169]. By contrast, a study investigating the sacroiliac biopsies of SpA patients with a relatively short symptom duration (7 years) and active sacroiliitis, of which the majority (56%) were diagnosed with AS, observed only a marginal number of (CD20^+^) B cells [170]. The inclusion of patients with early sacroiliitis, the pooling of SpA phenotypes, and a difference in immunohistological detection systems may explain the low number of B cells detected by this study [170].

In addition to the classical involvement of the axial skeleton, peripheral joints, such as the hip joints, may be affected in AS [171]. Synovial samples of the hip joints of AS patients that underwent total hip replacement surgery revealed the extensive infiltration of (CD20^+^) B lymphocytes, as well as (CD38^+^) IgG4-producing plasma cells, and ectopic lymphoid structures in the synovial membrane of the hip [172]. These lymphoid structures were organized and contained (CD21^+^) follicular dendritic cell networks, essential for the formation B cell follicles and germinal centers. An analysis of B cells infiltrating the synovial membrane of the hip B cells of an AS patient demonstrated that most B cells expressed immunoglobulins encoded by mutated variable (V) region genes, with mutational patterns with signs of antigen selection [173]. The V gene repertoire of the synovial B cells of this patient also provided some evidence for a biased repertoire of V genes used, compared to synovial B cells from the knee of a RA patient [173,174]. These findings suggest that antigen-specific B memory cells contribute to local immune response in the synovial membranes of AS patients.

Besides the hip joint, B cells were also observed in entheses of the hip and knee in SpA (almost all AS) patients [175]. Furthermore, inflamed synovial tissue from the peripheral joints (knee, wrist, or finger) of AS patients harbored B cells and plasma cells [176,177]. A histopathological analysis of synovial knee biopsies from anti-TNFα-treated SpA patients (50% diagnosed with AS) led to a reduction in the number of neutrophils, macrophages, and T cells, while B cell numbers and plasma cells remained stable [178]. As flaring may occur shortly after the discontinuation of TNFα blockers, Appel et al. suggested that residing B cells might be responsible for inciting relapses in AS [19]. Altogether, the infiltration of B cells and plasma cells in axial and peripheral joints, especially in a setting of inflammation, suggest the involvement of B cells at AS inflammatory sites.

## 7. Targeting B Cells in AS

The aforementioned findings, suggesting the possible role of B cells in the pathogenesis of AS, may provide a rationale for B-cell-depleting therapy. The most well-known and extensively studied anti-B cell therapy in various immune-mediated diseases is treatment with B-cell-depleting biological rituximab, targeting CD20-positive B cells [179]. There are a few small studies using rituximab in AS. The first successful treatment response was described in an anecdotal case study highlighting the efficacy of rituximab in an AS patient suffering from hepatitis B and naïve to anti-TNFα therapy [180]. Within the first two months after treatment, the patient showed a remarkable improvement, with significant reductions in BASDAI score, swollen joint count, ESR, and CRP [180]. Several other case reports revealed an adequate treatment response in patients with AS, especially patients naïve for anti-TNFα [181,182,183,184]. Nevertheless, not all AS patients respond to rituximab and, in some instances, flaring of the disease may occur during treatment with rituximab [181,185,186,187]. So far, the largest study was performed by Song et al., evaluating the effectiveness of rituximab (1000 mg) administered at baseline and week two during a twenty-four-week follow-up on 20 AS patients. These patients were divided into two groups, among which 10 AS patients were naïve for anti-TNFα therapy and the remainder exhibited an inadequate response to anti-TNFα therapy [22]. At week 24, a clinical response was observed in 50% of the AS patients belonging to the naïve treatment group, demonstrating an improvement of 20%, according to the Assessment of Spondyloarthritis International Society improvement criteria (ASAS20); ASAS40 was observed in 40% of the patients, and even 30% showed partial remission. This is only slightly lower than the improvement reported for TNFα inhibitor [188]. Notably, half of the patients naïve for TNFα antagonists exhibited a BASDAI50 response (i.e., a change in at least 50% of the BASDAI). By contrast, in the anti-TNFα failure group, 30% of the patients achieved an ASAS20 response and an ASAS40 response was observed only in one patient. At week 24, a total of nine patients (three anti-TNFα failures and six patients naïve for TNFα inhibitors) were good clinical responders in terms of an ASAS20 response [22,187]. In a follow-up study, five patients from among these clinical responders flared and received a second course of rituximab (1000 mg) [187]. Eight out of nine responders achieved an improvement of at least 50% in BASDAI, ASDAS, and CRP at 48 weeks [187]. Although no placebo-controlled trials using rituximab in AS have been performed thus far, the use of B cell depletion seems beneficial for a subgroup of patients, especially when naïve for TNFα inhibitors. Why AS patients need to be naïve for successful B cell depletion therapy is still enigmatic. Further research with larger groups of well-defined AS patients is required to determine which patients may benefit the most of anti-B cell therapy.

## 8. Conclusions

For a long time, the role of B cells in the pathogenesis of AS received little attention. However, in recent decades, a growing body of evidence has begun to point towards B cell involvement in the disease process, against prevailing dogma. In AS, the modulation of B cells is illustrated by genetic variation, along with the role of cytokines, such as IL-17 and IL-21, which may directly influence B cell function and development. Phenotypic analysis of peripheral blood B cells indicates that the frequencies of B cells with an activated phenotype and plasmablasts increase. The presence of a variety of autoantibodies to, for example, CD74/CLIP, and the proteins involved in bone homeostasis, as well as antibodies to microbial antigens, provides further evidence of B cell involvement in AS. The standardization of the detection methods for these novel (auto-) antibodies, and the evaluation of large, well-defined patient cohorts is warranted, however, to accurately evaluate the diagnostic and/or prognostic value of these antibodies. The presence of B cells, plasma cells, and ectopic lymphoid structures at classic inflammatory sites of AS is highly suggestive of active B cell participation in the disease process. A detailed analysis of B cells at inflammatory sites using -omics technologies may be used in the near future to obtain insights into their local role. Last, but certainly not least, B cell involvement in AS is further substantiated by the beneficial effects of B-cell-depleting therapy in a subcategory AS patients. These trials may also help to unravel the pathogenic contribution of B cells in AS.
